# RNA Interference Targeting Connective Tissue Growth Factor Inhibits the Transforming Growth Factor-****β****_**2**_ Induced Proliferation in Human Tenon Capsule Fibroblasts

**DOI:** 10.1155/2013/354798

**Published:** 2013-10-28

**Authors:** Jiaona Jing, Ping Li, Tiejun Li, Yuncheng Sun, Huaijin Guan

**Affiliations:** ^1^Eye Institute, Affiliated Hospital of Nantong University, 20 Xisi Road, Nantong, Jiangsu Province 226001, China; ^2^Nanjing Governmental Hospital, 116 Chengxian Street, Nanjing, Jiangsu Province 210018, China; ^3^Department of Life Science Center, Biomics Biotechnologies Co., Ltd., 76 Changxing Road, E&T Development Area, Nantong, Jiangsu Province 226016, China; ^4^Small RNA Technology and Application Institute, Nantong University, 76 Changxing Road, E&T Development Area, Nantong, Jiangsu Province 226016, China

## Abstract

*Purpose*. This study was to determine the effect of CTGF-small interfering RNA (siRNA) on TGF-**β**_2_-induced proliferation in human Tenon capsule fibroblasts (HTFs). *Methods*. HTFs were transfected with four of CTGF-siRNAs separately for screening of gene silencing efficacy that was determined by transcript level measured by quantitative real-time PCR (qRT-PCR). Recombinant TGF-**β**_2_ was added into the culture to stimulate the proliferation of HTFs. The gene silencing efficacy of the siRNAs was evaluated by qRT-PCR and immunofluorescence of CTGF transcript and protein levels. The viability of HTFs was determined by cell counting kit-8 (CCK-8). FCM was used to assess cell cycle after CTGF-siRNA transfection. *Results*. The expression of CTGF and proliferation of HTFs were increased significantly by TGF-**β**_2_ stimulation. The transfection of CTGF-siRNA abolished the upregulation of CTGF and cell proliferation induced by TGF-**β**_2_. The analysis of cell cycle indicated that CTGF-siRNA treatment stimulated cells from S phase to G0/G1 phase in comparison with the inverse physiologic function of TGF-**β**_2_. *Conclusion*. CTGF targeting siRNA could effectively suppress the expression of CTGF and attenuate the proliferation of HTFs. The siRNA approach may provide a therapeutic option for eliminating filtration bleb scarring after glaucoma filtration surgery (GFS).

## 1. Introduction

Glaucoma filtration surgery (GFS) is often required when medication fails to control intraocular pressure (IOP) adequately. Though this method has an immediate effect on reducing IOP, the long-term success is often impaired by the postoperative wound-healing process [1–3]. Previous studies have shown that human Tenon capsule fibroblasts (HTFs) located in the incision area play a major role in scar formation via the proliferation, migration, and synthesis of extracellular matrix (ECM) [4, 5]. Although antiscarring agents such as mitomycin C and 5-fluorouracil can prevent postoperative scarring and improve the success rate of trabeculectomy, their application is associated with relatively less specificity and an increased incidence of severe complications [6, 7].

Cytokines play crucial roles in scar formation of the bleb [8]. Among the cytokines, transforming growth factor-*β* (TGF-*β*) is an important profibrotic factor and is found in aqueous humor and other eye tissue [9–11]. TGF-*β*_2_ plays an important role in bleb scarring, which is one of the major reasons for the failure of GFS [12]. However, the completed suppression of TGF-*β* may result in significant adverse side effects because it plays broad physiological functions such as intercellular signaling and immune regulation [13]. Moreover, the existence of certain levels of antiproliferative mechanisms is required for homeostasis of epithelial cells and tumor suppression. Therefore, it is necessary to search for an alternative target for antifibrotic therapy after trabeculectomy.

Connective tissue growth factor (CTGF) is a secreted peptide which acts as a downstream mediator of TGF-*β*, and thus, also as a profibrotic factor [13]. Without blocking other physiological effects on TGF-*β* such as suppression on epithelial cells' growth and modulation of immune or inflammatory cells, inhibition of CTGF might specifically suppress the tissue scarring. In fibroblasts, CTGF is crucial in pathological fibrosis by promoting fibroblast proliferation, inducing ECM remodeling, and initiating myofibroblast differentiation [14, 15]. CTGF also stimulates chemotaxis and the expression of integrin, promotes endothelial cell growth, migration, adhesion and survival, and is thus implicated in endothelial cell function and angiogenesis [13]. The increase of CTGF expression has been proved to have contributed to many ocular fibrosis diseases including pterygium, cataract, and proliferative vitreoretinopathy [16–18]. 

RNA interference (RNAi) is an evolutionally conserved mechanism for regulating targeted gene expression [19]. RNAi is initiated by the conversion of double strain RNA into 21–23 nucleotide fragments, termed small interfering RNAs (siRNAs) [20]. In this process, siRNAs subsequently degrade their target mRNA in a sequence-dependence manner. Synthesized siRNA has been extensively used for manipulating gene expression *in vitro* and *in vivo *[20, 21]. The therapeutic application of siRNA has opened a new avenue for drug development for various diseases including ocular disorders [22, 23]. 

In this study, we tested the effect of synthesized CTGF-siRNA on the inhibition of CTGF expression and proliferation of HTFs stimulated by TGF-*β*_2_.

## 2. Material and Methods

### 2.1. Cell Culture and Identification

Human subconjunctival Tenon capsule samples were obtained from individuals undergoing strabismus surgery. The human tissue was used in strict accordance with the tenets of the Declaration of Helsinki, and institutional human experimentation committee approval was granted. Each donor signed informed written consent. The patients had no history of systemic or conjunctival diseases and did not take any topical ocular medications. HTFs were obtained as an expansion culture of the Tenon capsule explants of 1 × 1 cm^3^ and were propagated in Dulbecco's modified Eagle medium (DMEM, Invitrogen, Carlsbad, CA, USA) supplemented with 15% heat-inactivated fetal bovine serum (FBS, Hyclone, Logan, UT, USA), 100 U/mL penicillin, and 100 *μ*g/mL streptomycin (Sigma-Aldrich, Saint-Louis, Missouri, USA) in 5% CO_2_ humidified atmosphere at 37°C. HTFs of passage 3 to 6 were used in the experiments. Cells were identified by immunocytochemistry of fibroblast marker, vimentin, (monoclonal antivimentin from Santa Cruz, CA, USA) and epithelial cells marker, keratin (monoclonal antikeratin from Cell Signaling, Beverly, MA, USA).

### 2.2. CTGF-siRNA Sequences

siRNAs were derived from the coding region of the human CTGF gene (NM_001901). The design was based on the software (siRNA Target Finder) from Ambion (Austin, TX, USA), and the sequences were BLASTed against the Genbank for excluding potential homologs. The target sequences (5′ to 3′) and the duplexes of 4 relevant siRNAs are listed in Table 1. These siRNAs were synthesized and purified by Invitrogen (Carlsbad, CA, USA). In addition, a FAM-labeled nonspecific siRNA (Biomics, Nantong, China) was used for evaluating efficacy of transfection and as control siRNA as well.

### 2.3. siRNA Transfection and TGF-***β*_**2**_** Treatment

The cells were seeded in plates with a density of 4 × 10^5^ cells/mL in the complete culture medium without antibiotics. After 24 h, the culture media were then replaced with DMEM without both antibiotics and serum for 2 hours before transfection. The HTFs were transfected with CTGF-siRNA (50 nM) or control siRNA (50 nM) using Lipofectamine 2000 (Invitrogen, Carlsbad, CA, USA) following the manufacturer's protocol. After 24 h, the medium was replaced with the antibiotic- /serum-free DMEM with or without human TGF-*β*_2_ (5 ng/mL) (PeproTech, Rocky Hill, NJ, USA). The cells were harvested after 24 or 48 h of the treatment. The controls HTFs were either untreated or treated with Lipofectamine 2000 only.

### 2.4. Transfection Efficiency of siRNA

A FAM-labeled control siRNA (green fluorescence) was used for verifying transfection efficiency. The siRNA was transfected as described above. The transfection efficacy was evaluated by observation of the green fluorescence cells versus total cells using fluorescence microscope and flow cytometry (Becton, Dickinson and Company, Franklin Lakes, NJ, USA). The untreated HTFs were used as control. For flow cytometry, at least 1 × 10^4^ cells in each sample were analyzed. The experiments were repeated for at least 3 times.

### 2.5. Quantitative Real-Time PCR

Quantitative real-time PCR was used to determine the level of CTGF mRNA of HTFs after various treatments. Total RNA was isolated from HTFs using RISO reagent (Biomics, Nantong, China) and treated with DNase I. cDNA was synthesized by reverse transcriptase from total RNA with oligo-d (T) primers. Quantitative real-time PCR analysis was performed with the Bio-Rad IQ5 real-time PCR detection system (Bio-Rad, Hercules, CA, USA) using the SYBR Master mixture (Biomics, Nantong, China). The PCR reactions were performed in triplicate on each cDNA template along with triplicate reactions of a housekeeping gene, GAPDH. We used the following primers: for CTGF, forward (5′-ACTATGATTAGAGCCAACTG-3′) and reverse (5′-TGTTCTCTTCCAGGTCAG-3′); for GAPDH, forward (5′-GAAGGTGAAGGTCGGAGTC-3′) and reverse (5′-GAAGATGGTGATGGGATTTC-3′). The specific amplification was verified by melting curve analysis. The data were normalized against GAPDH. The expression levels were determined using the ΔΔCT method (IQ5 software version 2.0, Bio-Rad) and presented as fold changes. Experiments were performed in triplicate with 3 biological samples from each treatment.

### 2.6. Immunocytochemistry

HTFs were seeded in coverslips before transfection of siRNA. After being stimulated by TGF-*β*_2_ for 48 h, the cells on coverslips were washed three times with PBS and fixed with freshly prepared 4% paraformaldehyde solution in 0.01 M PBS for 30 min at room temperature. The fixed samples were incubated with primary antibodies: mouse monoclonal antivimentin (1 : 50 dilution), mouse monoclonal antikeratin (1 : 400 dilution), or mouse monoclonal anti-CTGF (1 : 100 dilution, Santa Cruz, CA, USA) overnight at 4°C in a humidified chamber. After being washed three times with PBS, the samples were further reacted with second antibodies: Alexa Fluor 488 goat anti-mouse (1 : 200 dilution, Invitrogen, Carlsbad, CA, USA) for 2 h at 37°C and counterstained with 5 *μ*g/mL of Hoechst 33342 (Sigma-Aldrich, Saint-Louis, Missouri, USA). The cells were viewed and photographed under a fluorescence microscope. 

### 2.7. CCK-8 Assay

The effect of CTGF-siRNA on HTFs viability after TGF-*β*_2_ treatment was determined by cell counting kit-8 (CCK-8, Biomics, Nantong, China) assay. This assay is based on the cleavage of the tetrazolium salt WST-8 by mitochondrial dehydrogenase in viable cells. After various treatments, HTFs in an exponential phase of growth were harvested and seeded in five 96-well plates at a density of 1 × 10^5^ cells/mL in a total volume of 100 *μ*L per well. After 0, 24, 48, 72, and 96 h of incubation, the viability of HTFs was analyzed by CCK-8 assay. The media were replaced by 100 *μ*L of DMEM containing CCK-8 (10 *μ*L) to each well. After 3.5 h of incubation at 37°C, the absorbance at 450 nm was measured with a Thermomax microplate reader. The experiment was repeated three times.

### 2.8. Flow Cytometry

After being transfected with siRNA and treated with TGF-*β*_2_ for 48 h, cell cycle was checked by flow cytometry. The HTFs were collected by trypsinization and washed twice with PBS before being resuspended at 1 × 10^6^ cells/mL in PBS and fixed in 70% ice-cold ethanol (v/v) overnight at 4°C. Fixed cells were stained with 0.5 mL of propidium iodide (Sigma-Aldrich, Saint-Louis, Missouri, USA)/RNase staining buffer (BD Pharmingen, San Diego, CA, USA) in the dark at 4°C for 30 min. The numbers of cells at G0/G1, S, and G2/M fractions were analyzed using a flow cytometer (BD FACSCalibur, BD Bioscience, USA). Proliferation index was calculated according to PI = (G2/M + S)/(G0/G1 + S + G2/M).

### 2.9. Statistical Analysis

Statistical analysis was performed using SPSS software (SPSS V 14.0; SPSS Inc). All results are presented as the mean ± SD. One way ANOVA was performed for comparing the differences among groups. Differences with *P* < 0.05 were considered statistically significant.

## 3. Results

### 3.1. Identification of Human Tenon Capsule Fibroblasts

**Figure 1:**
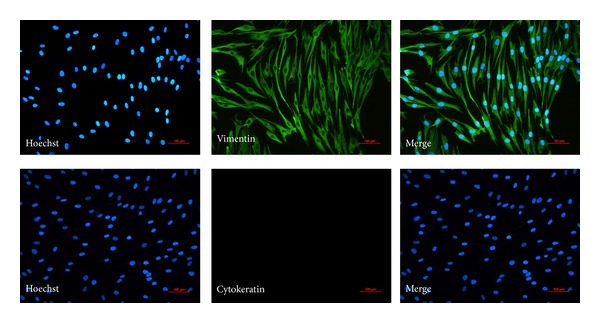
Identification of human Tenon capsule fibroblasts. A vimentin and cytokeratin immunostaining technique was used to detect fibroblast feature of the cultured cells. Fibroblast produced vimentin constitutively with the cytoplasm staining positively (in green). But cytokeratin staining in the fibroblast is negative. Nuclei stained with Hoechst were seen in blue.

Vimentin and keratin are cell surface markers for fibroblast and epithelium respectively. The cultured cells were stained positive for vimentin and negative for keratin (Figure 1). The results excluded the possible contamination of conjunctival epithelia during the cell culture.

### 3.2. Transfection Efficiency of siRNA

**Figure 2:**
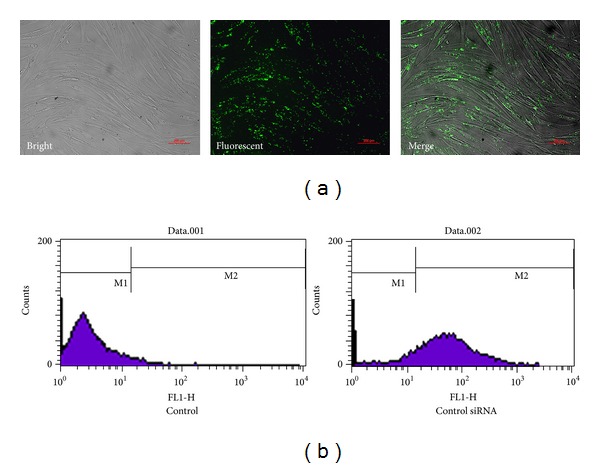
Transfection efficiency of siRNA. (a) Transfection efficiency of HTFs transfected with FAM-labeled control siRNA was observed by a fluorescence microscope. Green staining in cells stands for effective transfection. (b) FCM was used to analyze the transfection efficiency of siRNA. HTFs transfected with/without control siRNA were counted by FCM. Untransfected cells were marked with M1, and FAM-labeled cells were marked with M2 (here we just show one of the results).

The results indicated that most HTFs displayed green fluorescence after the transfection of FAM-labeled control siRNA (Figure 2(a)). HTFs showed the highest transfection efficiency of siRNA by being observed under fluorescence microscopy. The transfection was efficient in that 83.7% of the cells displayed green fluorescence detected by FCM (data not shown) (Figure 2(b)). The transfection efficiency implied that Lipofectamine 2000 could effectively introduce siRNA into HTFs.

### 3.3. Suppression of CTGF mRNA Expression

**Figure 3:**
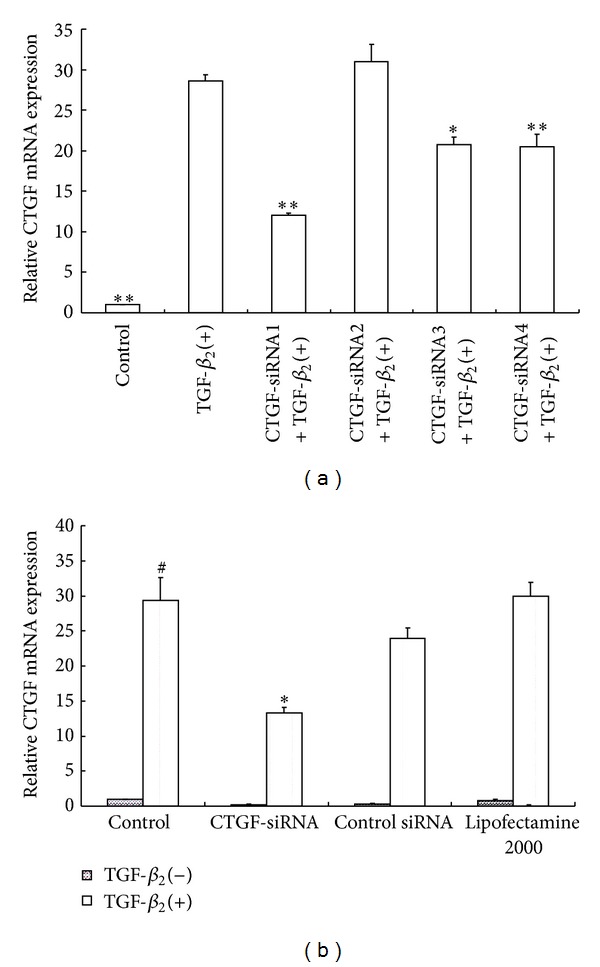
siRNA inhibition of CTGF mRNA expression. Serum starved HTFs were transfected with CTGF-siRNAs (siRNA1–siRNA5) or control siRNA, respectively, before being stimulated with TGF-*β*_2_ for 24 h. (a) Comparison of relative expression of CTGF mRNA in cultured HTFs transfected with different siRNAs. Data were from three experiments. *P < 0.05, **P < 0.01 versus TGF-*β*_2_(+). (b) Comparison of transcription levels of CTGF in HTFs under different conditions. Data were from three experiments. ^#^ *P* < 0.01 versus HTFs stimulated without TGF-*β*_2_ in control group. *P < 0.01 versus HTFs treated with TGF-*β*_2_ only.

After TGF-*β*_2_ induction, the HTFs transfected with CTGF-siRNA1, CTGF-siRNA3, or CTGF-siRNA4 but not CTGF-siRNA2 demonstrated the reduced CTGF gene expression. A 57.9% reduction in CTGF transcript level was observed after being transfected with CTGF-siRNA1 (*P* < 0.01), while CTGF-siRNA3 and CTGF-siRNA4 caused 27.3% (*P* < 0.05) and 28.4% (*P* < 0.01) reductions of the CTGF transcript levels, respectively, (Figure 3(a)) in comparison with that from HTFs without transfection. Therefore, CTGF-siRNA1 was used in follow-up experiments, named CTGF-siRNA. The CTGF mRNA level increased significantly after TGF-*β*_2_ treatment for 24 h compared with that of TGF-*β*_2_(−) group (*P* < 0.01, Figure 3(b)). There was no significant difference among the control siRNA group, Lipofectamine 2000 group, and the control group stimulated with TGF-*β*_2_ (Figure 3(b)).

### 3.4. Suppression of CTGF Protein Expression

**Figure 4:**
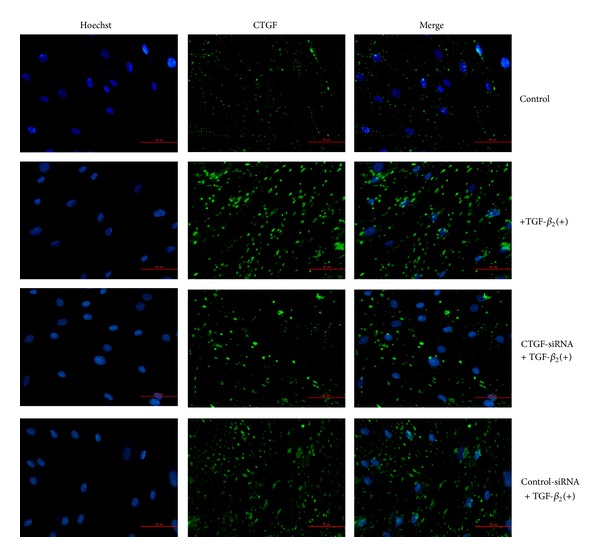
Suppression of CTGF protein expression in HTFs by siRNA. HTFs were stimulated with TGF-*β*_2_ for 48 h after cells were transfected with CTGF-siRNA or control siRNA. Immunofluorescence analysis of HTFs was performed to visualize the CTGF protein in cell matrix (in green) after various treatments. Nuclei stained with Hoechst were seen in blue.

 The effect of the CTGF-siRNA on expression of CTGF protein was determined by immunocytochemical staining. As shown in Figure 4, control HTFs exhibited a weak green punctiform staining in the cytoplasm. After treatment with TGF-*β*_2_, a distinguished strong pattern of punctuate patches of staining was displayed in cells, indicating enhanced CTGF expression. The treatment of CTGF-siRNA with the TGF-*β*_2_ stimulated cells led to a considerable reduction of fluorescence staining intensity compared with that of TGF-*β*_2_(+) group. HTFs treated with control siRNA exhibited a similar staining intensity and pattern as that of the TGF-*β*_2_ treated cells.

### 3.5. CTGF-siRNA Inhibits Viability of HTFs

**Figure 5:**
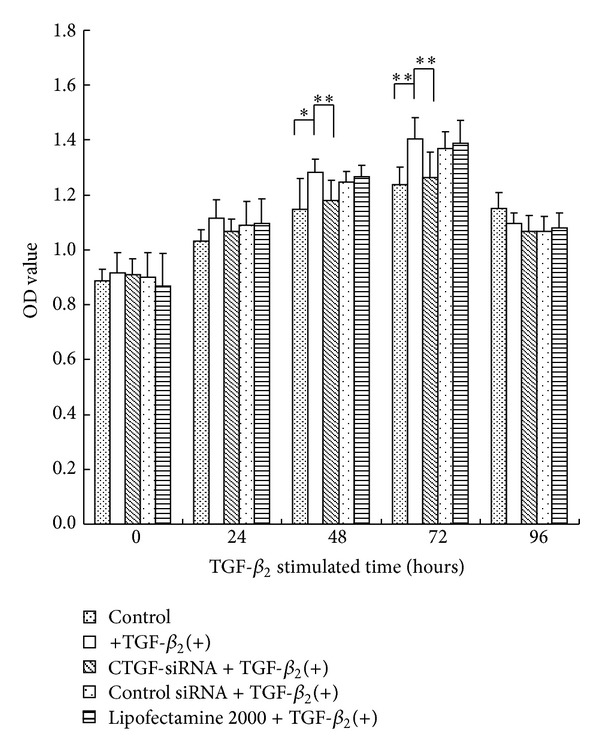
CTGF-siRNA reduces the viability of HTFs. Serum starved HTFs were transfected withCTGF-siRNA, control siRNA, or Lipofectamine 2000 before being stimulated with TGF-*β*_2_ for 0, 24, 48, 72, and 96 h. The viability of HTFs was analyzed by CCK-8 assay. CTGF-siRNA suppressed the viability of TGF-*β*_2_ stimulated cells at the time points of 48 h and 72 h, respectively. Data were from three experiments. *P < 0.05, **P < 0.01.

The viability of HTFs was detected by CCK-8. As shown in Figure 5, the cell growth showed that exogenous TGF-*β*_2_ might offer a growth advantage for HTFs. In contrast to only TGF-*β*_2_ stimulation group, the CTGF-siRNA treatment reduced the viability of TGF-*β*_2_ stimulated cells by 7.88% (*P* < 0.01) and 10.11% (*P* < 0.01) at the time points of 48 h and 72 h, respectively. After TGF-*β*_2_ treatment, the cell viability of HTFs treated with control siRNA or Lipofectamine 2000 was similar to that of TGF-*β*_2_-treated cells, indicating a low cytotoxicity by Lipofectamine 2000. There was no significant difference in HTFs viability between the TGF-*β*_2_(+) group and the CTGF-siRNA group (*P* > 0.05) at the time points of 24 h and 96 h. This indicated that CTGF-siRNA could effectively inhibit the proliferation of HTFs at the time points of 48 h and 72 h.

### 3.6. Effect of CTGF-siRNA on Cell Cycle

The effect of CTGF-siRNA on the cell cycle was evaluated by flow cytometry (Table 2). The percentage of HTFs in G0/G1 phase in the TGF-*β*_2_(+) group (88.290 ± 0.335%) was significantly reduced compared with the control group (94.917 ± 1.063%) (*P* < 0.01) and was higher in the CTGF-siRNA group (91.177 ± 1.064%) than the TGF-*β*_2_(+) group (*P* < 0.05). On the contrary, the percentage of HTFs in S phase in the TGF-*β*_2_(+) group (9.037 ± 0.258%) was increased compared with the control group (1.613 ± 0.372%) (*P* < 0.01) and was lower in the CTGF-siRNA group (5.410 ± 0.589%) than the TGF-*β*_2_(+) group (*P* < 0.05). There was no significant difference between the TGF-*β*_2_(+) group and the control siRNA group in G0/G1 phase or S phase (*P* > 0.05). 

**Figure 6:**
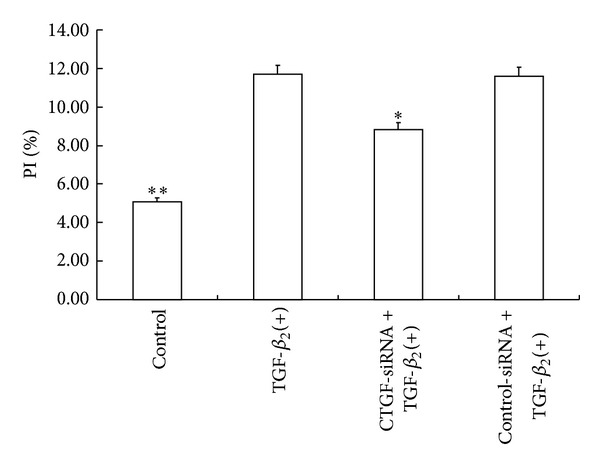
CTGF-siRNA decreases proliferation index of HTFs. HTFs were stimulated with TGF-*β*_2_ for 48 h after cells were transfected with CTGF-siRNA or control siRNA. PI of HTFs was calculated according to cell cycle analyzed by flow cytometry. CTGF-siRNA decreased the PI of TGF-*β*_2_ treated cells. Data were from three independent experiments. *P < 0.05, **P < 0.01 versus TGF-*β*_2_(+) group.

Flow cytometry showed that the cells treated with TGF-*β*_2_ had a higher value in proliferation index (PI) than the control group (*P* < 0.01) (Figure 6). However, the pretreatment with CTGF-siRNA decreased the PI of TGF-*β*_2_ treated cells (*P* < 0.05). 

## 4. Discussion

The scar formation after GFS is consistent with the production of connective tissue during wound repairing. TGF-*β* is known to be the most potent growth factor involved in wound healing and also a key modulator in the process of bleb fibrosis [24–26]. There are three isoforms of TGF-*β* in human, and the level of TGF-*β*_2_ is the highest in aqueous humor and other eye tissues. After filtering operations, aqueous humor comes into direct contact with the connective tissue of the subconjunctiva and stimulates fibroblasts proliferation. This might be responsible for the failure of trabeculectomy. Our study shows that HTFs treated with TGF-*β*_2_ had increased viability. These cells also had an increased portion in S phase, a decreased portion in G0/G1 phase, and higher value in PI than the control group. These results indicated that TGF-*β*_2_ could promote the proliferation of HTFs significantly. Recent studies have proved that treating TGF-*β*_2_ with monoclonal antibodies or antisense nucleotides could inhibit fibroblast proliferation and prolong the survival of experimental filtering blebs in animal models [27, 28]. 

Researches have suggested that CTGF may mediate the key actions of TGF-*β* in scar formation, such as stimulation of cell proliferation, extracellular matrix protein synthesis, and myofibroblast differentiation in fibroblasts [29–32]. Blockade of CTGF expression or its function may effectively inhibit the effects of TGF-*β*. Treating CTGF with antisense oligonucleotides or neutralizing antibodies could decrease TGF-*β*-mediated collagen synthesis in human corneal fibroblast. CTGF antisense oligodeoxynucleotide could inhibit TGF-*β*_1_-mediated myofibroblast differentiation and corneal-fibroblast-seeded collagen lattices (FSCL) contraction [33, 34]. In our study, we further illustrated that siRNA targeting CTGF could attenuate the proliferation of HTFs. 

Double-stranded siRNA is an effective approach to induce gene silencing in cells [35]. Inhibition of gene expression through siRNA is superior to conventional gene-blocking approaches due to the following reasons: (1) inhibitory effect is more potent and stable [36, 37]; (2) targeting of gene expression is more specific [38]; (3) blocking efficacy can be passed on for multiple generations [37]. Therefore, there are more potential clinical applications for siRNA [35]. Previous reports have shown that TGF-*β*_2_ coupled with CTGF mediated the bleb-scarring process [8, 27, 39]. In the present study, we treated the normal HTFs with exogenous TGF-*β*_2_ to simulate cell proliferation that mimic bleb formation after filtration surgery. We came to a conclusion that TGF-*β*_2_ could increase the expression of CTGF in HTFs and this effect could be abolished by pretreatment with CTGF-siRNA. 

The induction of proliferation by CTGF has been found in some mesenchymal cells [13]. Ishibuchi et al. demonstrated that the proliferation was constantly suppressed by CTGF-silencing in normal and systemic sclerosis fibroblast [40]. Another study also showed that CTGF induced cornea stroma fibroblasts proliferation [41]. In our study, the analysis of cell cycle revealed that CTGF-siRNA treatment resulted in an increased proportion in G0/G1 phase and an inverse one in S phase. The reduction of the viability of HTFs was also detected by CCK-8 assay. These results suggested that downregulation of CTGF expression could induce the cell cycle of HTFs to arrest in G0/G1 phase and might prevent its DNA synthesis, which might be the mechanism of inhibition of cell proliferation after transfection of siRNA-CTGF in HTFs. Some studies have also suggested that reduction of ECM accumulation may attenuate cell proliferation. To validate this hypothesis, the effect of CTGF-siRNA on ECM in HTFs and the relationship between ECM and proliferation are needed to be conducted.

## 5. Conclusions

In summary, we showed that siRNA targeting CTGF could be successfully transfected into HTFs *in vitro* and could subsequently inhibit the proliferation of HTFs. These results suggested that specific inhibitors of CTGF could have beneficial effects on preventing pathogenic fibrosis in bleb after glaucoma filtration surgery. 

## Figures and Tables

**Table 1:** Targets and duplex sequences for human CTGF specific siRNAs and control siRNA.

siRNA duplex	CTGF target sequence(5′-3′)	siRNA duplex sequences
CTGF-siRNA1	(1024)GCACCAGCATGAAGACATACC	5′-GCACCAGCAUGAAGACAUACCdTdT-3′
		5′-GGUAUGUCUUCAUGCUGGUGCdTdT-3′
CTGF-siRNA2	(862)CCCGGGTTACCAATGACAACG	5′-CCCGGGUUACCAAUGACAACGdTdT-3′
		5′-CGUUGUCAUUGGUAACCCGGGdTdT-3′
CTGF-siRNA3	(883)CCTCCTGCAGGCTAGAGAAGC	5′-CCUCCUGCAGGCUAGAGAAGCdTdT-3′
		5′-CCAAGCCUAUCAAGUUUGAGCdTdT-3′
CTGF-siRNA4	(994)CCAAGCCTATCAAGTTTGAGC	5′-CCAAGCCUAUCAAGUUUGAGCdTdT-3′
		5′-GCUCAAACUUGAUAGGCUUGGdTdT-3′
control siRNA		5′-UUCUCCGAACGUGUCACGUdTdT-3′
		5′-ACUCCUCGCAGCAUUUCCCGGdTdT-3′

Four siRNAs were designed from the coding sequence of human CTGF gene. The target sequences (5′-3′) and the siRNA duplex sequences are listed, with the position of the first nucleotide in CTGF sequence shown in parentheses. A nonspecific, scrambled siRNA duplex as control siRNA was used as a control.

**Table 2:** Effect of CTGF-siRNA on cell cycle of HTFs.

Group	G0/G1 (%)	S (%)	G2/M (%)
Control	94.917 ± 1.063	1.613 ± 0.372	3.470 ± 1.131
TGF-*β*_2_(+)	88.290 ± 0.335*	9.037 ± 0.258*	2.673 ± 0.153
CTGF-siRNA + TGF-*β*_2_(+)	91.177 ± 1.064^#^	5.410 ± 0.589^#^	3.413 ± 0.533
Control siRNA + TGF-*β*_2_(+)	88.390 ± 1.074	9.047 ± 0.284	2.563 ± 0.825

Serum starved HTFs were transfected with CTGF-siRNA or control siRNA before being stimulated with TGF-*β*_2_ for 48 h. Flow cytometry was used to analyze the effect of CTGF-siRNA on cell cycle (G0/G1, S, G2/M phase) after various treatments. Data were from three experiments. *P < 0.01 versus control group, ^#^ *P* < 0.05 versus TGF-*β*_2_(+) group.
